# Structural determinants of 5′,6′-epoxyeicosatrienoic acid binding to and activation of TRPV4 channel

**DOI:** 10.1038/s41598-017-11274-1

**Published:** 2017-09-05

**Authors:** Alejandro Berna-Erro, Mercè Izquierdo-Serra, Romina V. Sepúlveda, Fanny Rubio-Moscardo, Pau Doñate-Macián, Selma A. Serra, Julia Carrillo-Garcia, Alex Perálvarez-Marín, Fernando González-Nilo, José M. Fernández-Fernández, Miguel A. Valverde

**Affiliations:** 1 Laboratory of Molecular Physiology, Dept. of Experimental and Health Sciences, Universitat Pompeu Fabra, Barcelona, Spain; 2 Universidad Andrés Bello, Center for Bioinformatics and Integrative Biology, Facultad de Ciencias Biológicas, Av. República 239, Santiago, Chile; 3 Unitat de Biofísica, Centre d’Estudis en Biofísica, Departament de Bioquímica i de Biologia Molecular, Universitat Autònoma de Barcelona, 08193 Bellaterra, Spain; 4 Centro Interdisciplinario de Neurociencia de Valparaíso, Facultad de Ciencias, Universidad de Valparaíso, Valparaíso, 2366103 Chile

## Abstract

TRPV4 cation channel activation by cytochrome P450-mediated derivatives of arachidonic acid (AA), epoxyeicosatrienoic acids (EETs), constitute a major mechanisms of endothelium-derived vasodilatation. Besides, TRPV4 mechano/osmosensitivity depends on phospholipase A_2_ (PLA_2_) activation and subsequent production of AA and EETs. However, the lack of evidence for a direct interaction of EETs with TRPV4 together with claims of EET-independent mechanical activation of TRPV4 has cast doubts on the validity of this mechanism. We now report: 1) The identification of an EET-binding pocket that specifically mediates TRPV4 activation by 5′,6′-EET, AA and hypotonic cell swelling, thereby suggesting that all these stimuli shared a common structural target within the TRPV4 channel; and 2) A structural insight into the gating of TRPV4 by a natural agonist (5′,6′-EET) in which K535 plays a crucial role, as mutant TRPV4-K535A losses binding of and gating by EET, without affecting GSK1016790A, 4α-phorbol 12,13-didecanoate and heat mediated channel activation. Together, our data demonstrates that the mechano- and osmotransducing messenger EET gates TRPV4 by a direct action on a site formed by residues from the S2-S3 linker, S4 and S4-S5 linker.

## Introduction

The transient receptor potential vanilloid 4 (TRPV4) is a widely expressed nonselective cation channel that shows a polymodal gating behavior^1, 2^. TRPV4 is activated by physical stimuli such as hypotonicity^3–5^, mechanical forces^6–8^, moderate heat^9–11^ or UVB radiation^12^, and by both natural (epoxyeicosatrienoic acids, EETs^13, 14^ and bisandrographolide^15^) and synthetic agonists (e.g., 4α-phorbol 12,13-didecanoate (4α-PDD)^16^ and GSK1016790A^17^). Due to this gating promiscuity, TRPV4 participates in multiple physiological processes, including cellular^5, 18^ and systemic volume homeostasis^19, 20^, endothelial function and angiogenesis^14, 21–23^, epithelial hydroelectrolyte transport^24^, nociception^25^, bladder voiding^26^, ciliary beat frequency regulation^8, 27^, innate immunity^28^, matrix stiffness^29^, cartilage maintenance and chondroprotection^30, 31^, and bone development^32^.

Intracellular lipid metabolites are important modulators of TRPV4 gating: Phosphatidylinositol 4,5-bisphosphate (PIP_2_) binding to a stretch of positive charges within the N-tail of each cannel subunit is required for TRPV4 activation by hypotonicity and heat^11^ while EETs derived from AA promote TRPV4 opening^13^. EETs also appear to act as messengers that mediate TRPV4 activation in response to either hypoosmotic shock^33^ or mechanical stimulation^8, 34^. In this regard, PLA_2_ is activated by hypotonic and mechanical stimulation^35, 36^ but no direct measurements of EETs have been reported in response to these stimuli. Besides, EETs constitute a major type of endothelium-derived hyperpolarizing factors that promote vascular relaxation through two plausible mechanisms involving TRPV4. First, EETs induce TRPV4-mediated Ca^2+^ influx into smooth muscle cells that ends in the activation of large conductance Ca^2+^-gated K^+^ (BK_Ca_) channels, resulting in direct smooth muscle hyperpolarization and vasodilation^14, 37^. Second, autocrinally released EETs promote TRPV4-mediated Ca^2+^ entry in endothelial cells^21^ that stimulates the activity of small and intermediate conductance Ca^2+^-gated K^+^ (SK_Ca_ and IK_Ca_) channels, causing endothelial-dependent vascular relaxation^38–40^. Moreover, activation of the cerebrospinal fluid Na^+^ level sensor Na_X_ in brain glial cells produces EETs that lead to the activation of TRPV4-positive neurons in sensory circumventricular organs to induce water intake^41^.

EETs are also modulators of other ion channels such as the ATP-sensitive K^+^ channel Kir6.2^42^, the BK_Ca_ channel^43^, L-type voltage-gated Ca^2+^ channels^44^ or the epithelial Na^+^ channel ENaC^45^. Depending on the channel, the effect of EETs is produced either by their direct interaction with a specific channel site^42^ or through different intracellular signaling pathways^43–45^. However, despite the physiological relevance of TRPV4 modulation by EETs, it is still unknown how EETs ultimately activate TRPV4. We now combine molecular simulations along with binding assays and functional studies to provide strong evidences supporting that EET-induced TRPV4 gating is due to direct EET binding to a crevice formed by helical segments S1 through S4 of each TRPV4 subunit, with a critical role of the K535 residue located at the S2-S3 linker in the stabilization of the ligand position.

## Results

**Figure 1:**
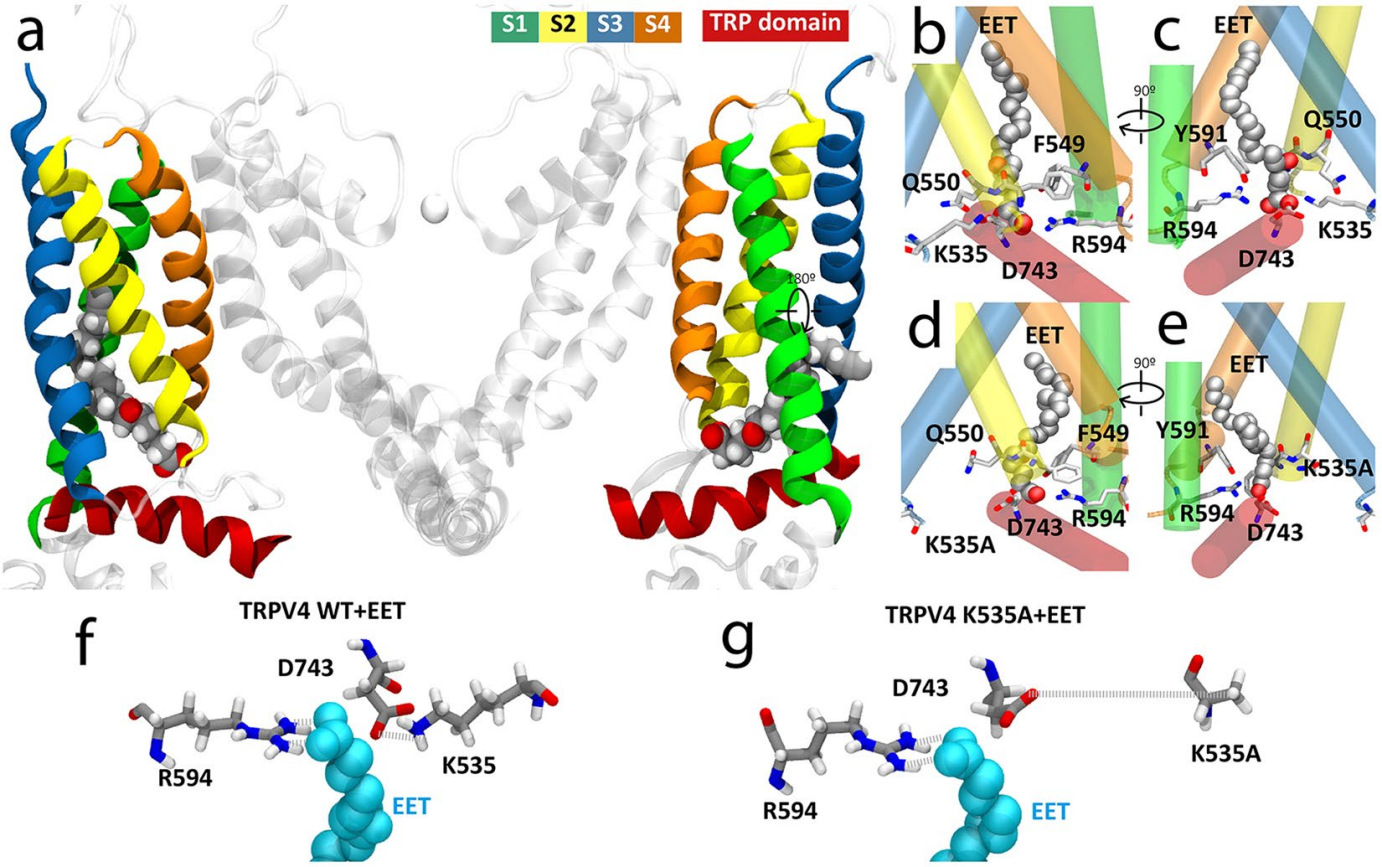
Predicted model of TRPV4 channel interaction with 5′,6′-EET. (**a**) TRPV4 structural model showing only two of the four identical subunits (side view). Color-coded transmembrane segments S1-S4, TRP box and EET molecule are highlighted. Images of the predicted EET-binding site in TRPV4-WT (**b**,**c**) and TRPV4 K535A (**d,e**) systems. Residues showing more than 60% of time occupancy are displayed in licorice representation. K535A residue from TRPV4 K535A is displayed in licorice representation even though its interaction is not significant. (**f,g**) Bottom view detail of the interaction of 5′,6′-EET with residues K535 and R594 in the TRPV4-WT (**f**) and TRPV4-K535A (**g**) systems. Note the loss of interaction between mutated residue A535 and both D743 and EET molecule.

To gain structural insights into the potential direct interaction of 5′,6′-epoxyeicosatrienoic acid (5′,6′-EET) with the TRPV4 channel we combined *in silico* molecular docking with molecular dynamics (MD) simulations. Docking poses clustered in 4 cavities in TRPV4, representing a unique binding site defined in the TRPV4 four-fold symmetry. The TRPV4 residues within 5 Å of the 100 docking poses are highlighted in the sequence shown in Supplementary Fig. 1. The docking energy ranged from −6.0 to −7.2 kcal/mol. This initial docking was further refined using smaller docking boxes (20 Å × 20 Å × 20 Å) around the defined binding site, to obtain lower binding energy values. The docking solution presented (Supplementary Fig. 2) represents the docking pose (out of 10) with the lowest energy (−7.4 kcal/mol), where TRPV4 residues within a distance of 3.5 Å of 5′,6′-EET are indicated. To evaluate the stability of the configuration determined by the *in silico* docking and further explore the interactions between 5′,6′-EET and TRPV4, a 120 ns MD simulation (Fig. 1a) was performed based on a previously reported TRPV4 model^46^ and using the configuration shown in Supplementary Fig. 2 as starting point. Based on the most frequent interactions, i.e., residues showing more than 60% of time occupancy (displayed in licorice representation in Fig. 1b,c), MD simulation identified a 5′,6′-EET binding site in the TRPV4-WT system conformed by residues from S2-S3 linker (K535, F549 and Q550), S4 (Y591) and S4-S5 linker (R594) (Fig. 1a–c, f and Supplementary Fig. 3) that were also predicted by the *in silico* docking. These residues, which are conserved throughout evolution (Supplementary Fig. 4), re-direct the 5′,6′-EET′s aliphatic chain upwards. Among those residues, amino acids K535 and R594 seem to play a crucial role as they face the 5′,6′-EET carboxylic group to stabilize ligand position (Fig. 1f and Supplementary Fig. 3). Residue K535 also generates a salt bridge with D743 in the TRP box (Fig. 1f).

In order to test the relevance of these two residues in 5′,6′- EET binding we set a MD simulation in which 5′,6′-EET was added to a channel with K535 mutated to A535 (TRPV4-K535A). The 120 ns simulation reported the loss of the interaction between 5′,6′-EET and residue A535 (Fig. 1d,e) as well as the salt bridge between residues 535 and 743 (Fig. 1g). All these facts suggest a loss of stability of the 5′,6′-EET position in the TRPV4-K535A system, which correlates with the values of the root-mean square deviation of atomic positions (RMSD) calculated in both systems: TRPV4-WT + EET, 3.4 ± 0.5 Å and TRPV4-K535A + EET, 4.2 ± 0.8 Å (mean ± S.D., n = 4). Molecular dynamic simulations using TRPV4-K535Q and TRPV4-R594Q systems (to neutralize charges at those residues) were also run and, similar to the TRPV4-K535A, demonstrated loss of stability of the 5′,6′-EET position in these two systems (Supplementary Fig. 3). In the absence of 5′,6′-EET no lipids were observed in the EET binding pocket of the TRPV4-WT or TRPV4-K535A systems (Supplementary Fig. 5).

**Figure 2:**
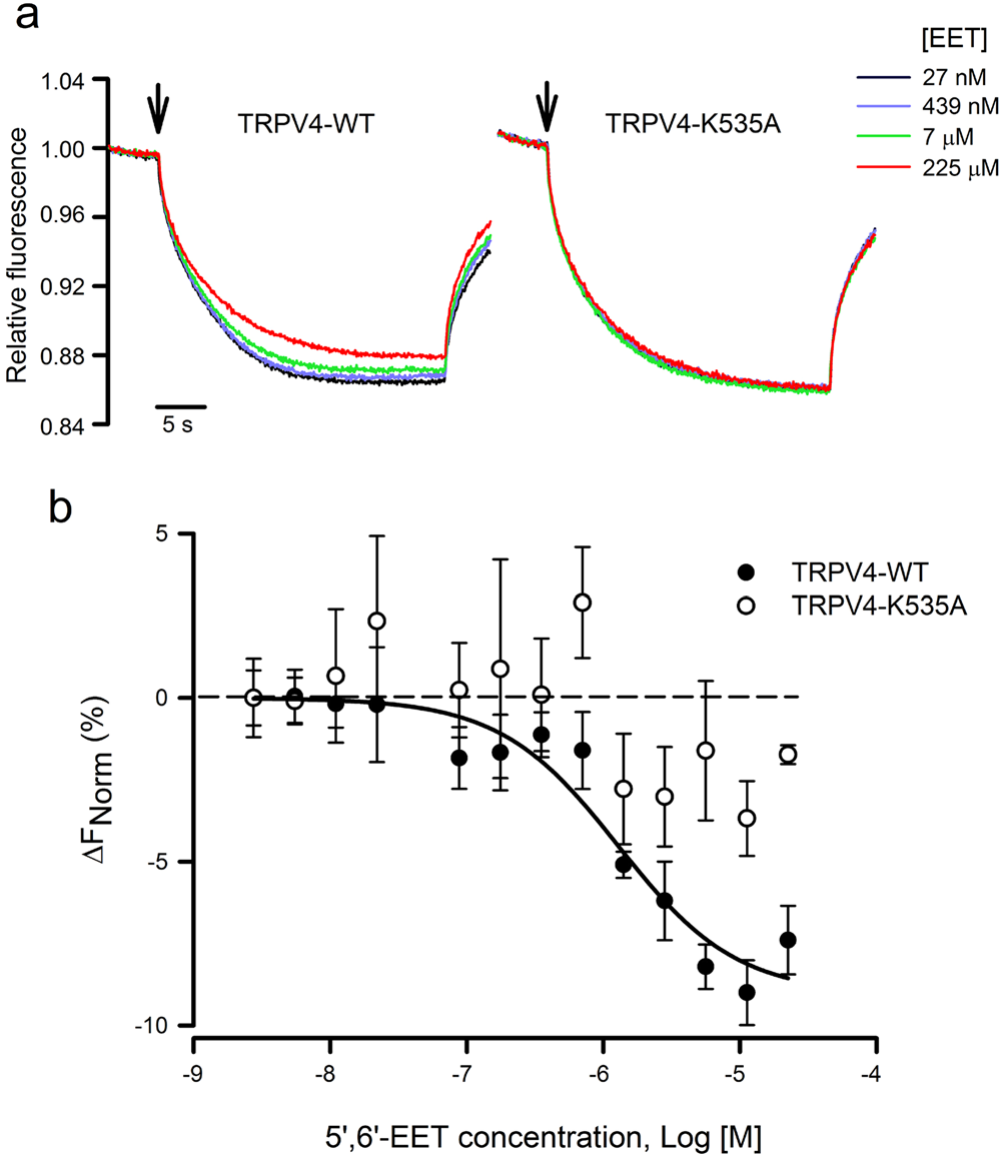
Thermophoretic analysis of the TRPV4-EET interaction. (**a**) Typical signal of a microscale thermophoresis (MST) experiment showing the thermophoretic movement (induced by infrared laser activation, arrow) of the GFP-labeled TRPV4 WT or K535A mutant channel and the subsequent fluorescence change measured for 30 s in the presence of increasing concentrations of 5′,6′-EET, from 27 nM (blue trace) to 225 µM (red trace). GFP-TRPV4 channels and 5′,6′-EET were incubated for 30–60 min in PBS, with 2% ethanol as vehicle. **(b)** Average changes in normalized fluorescence obtained for GFP-labeled TRPV4 WT (n = 4) and K535 (n = 4) channels plotted against 5′,6′-EET concentration and fitting of the TRPV4-WT data.

In order to test the physical interaction between 5′,6′-EET and TRPV4 and to evaluate the predicted relevance of residue K535, we used the microscale thermophoresis (MST) assay. MST records the migration behavior of fluorescently labeled proteins in a microscopic temperature gradient, which is influenced by any interacting molecule. Thus, fluorescence changes in a heated spot of the GFP-tagged TRPV4 protein containing solution are measured as a function of increasing concentration of the non-fluorescent 5′,6′-EET, allowing the estimation of *K*_*d*_ values. Figure 2a shows fluorescence traces obtained for TRPV4-WT-GFP and TRPV4-K535A-GFP proteins at four representative 5′,6′-EET concentrations. The change in thermophoresis, expressed as the change in the normalized fluorescence (ΔF_Norm_), upon increasing concentrations of 5′,6′-EET yielded a binding curve for TRPV4-WT-GFP with a *K*_d_ of 12.8 ± 4 μM (Fig. 2b). In the case of TRPV4-K535A, data did not fit to a binding curve in the range of agonist concentration tested, suggesting the lack of interaction between 5′,6′-EET and the mutant channel (Fig. 2b).

Residue R594 has been previously implicated in general channel gating as its mutation prevents channel activity in response to many different stimuli^47^, an observation we also report now (Supplementary Fig. 6) that precludes further functional studies on the role of this residue in the interaction of EET with the TRPV4 protein. Nevertheless, the relevance of residue R594 to the TRPV4 interaction with EET was also evaluated by MST assays (Supplementary Fig. 7). GFP and TRPV4–R594A-GFP proteins showed similar MST responses. Maximal ΔF_Norm_ values obtained with GFP and TRPV4–R594A-GFP proteins were significantly smaller than those obtained with TRPV4-WT-GFP.

**Figure 3:**
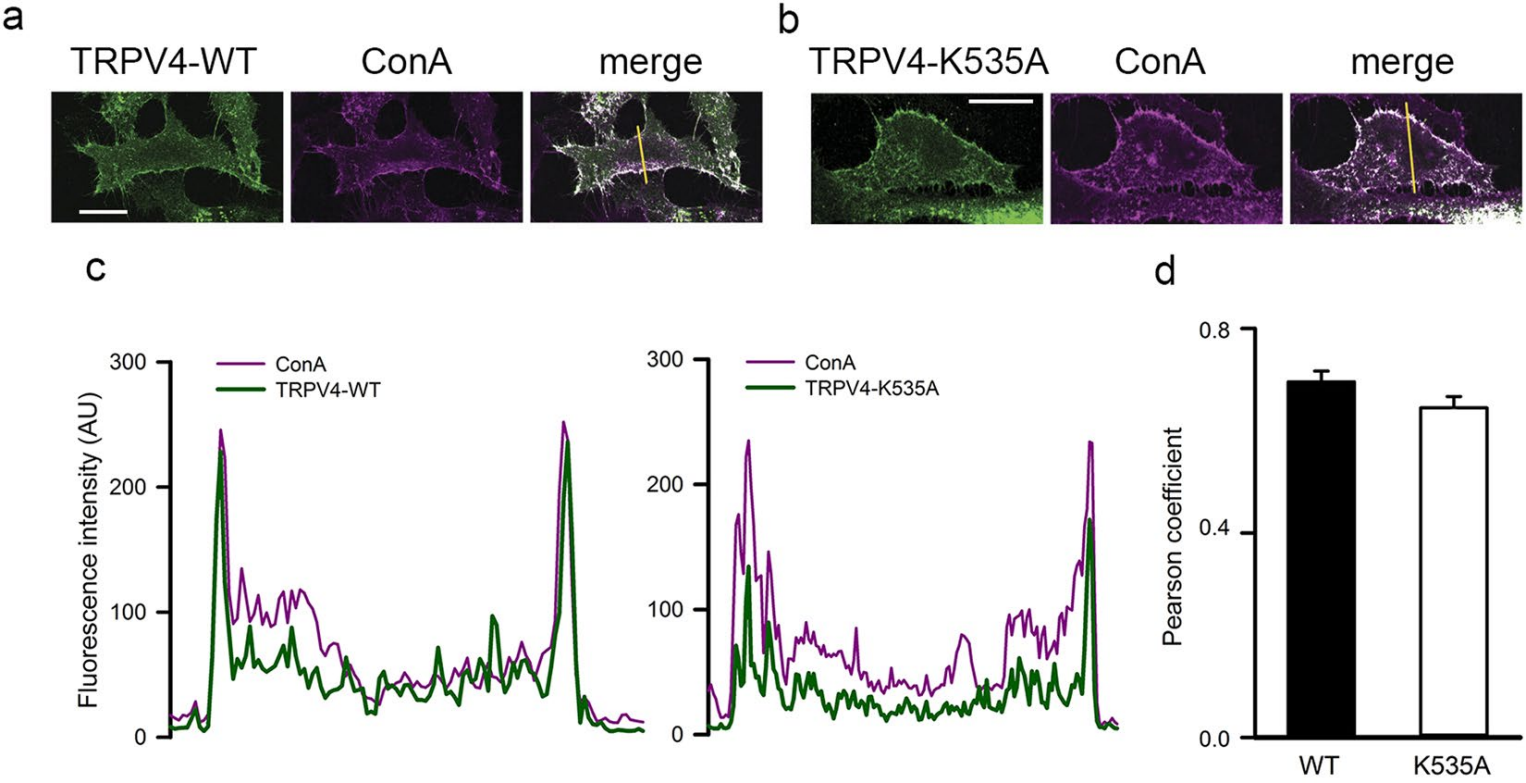
Mutation K535A does not alter membrane localization of heterologously expressed TRPV4 channels. Co-localization of TRPV4-WT (**a**) and TRPV4-K535A (**b**) channels (green) expressed in HeLa cells with the plasma membrane marker concanavalin A (magenta). Co-localized pixels are shown in white in the merge panels. The yellow line on the merge panels indicates the plot profile analysis (**c**) performed on each image using ImageJ software. Scale bar = 20 μm. (**d**) Pearson correlation coefficients obtained with the plot profile analysis of TRPV4-WT and TRPV4-K535A channels in the plasma membrane location. Mean ± S.E.M., P = 0.1 Mann-Whitney.

Both the molecular dynamics and MST data indicated that the TRPV4-K535A channel has lost its ability to bind 5′,6′-EET. To test whether this was also translated to an impaired channel response to EET we combined calcium imaging and patch-clamp techniques to evaluate channel activity. First, we showed that TRPV4-WT (Fig. 3a) showed similar pattern of expression than TRPV4-K535A channel (Fig. 3b) as well as co-localization to the plasma membrane, identified with the plasma membrane marker concanavalin A in transfected HeLa cells. Both TRPV4–WT and TRPV4–K535A channels showed similar overlapping plot profiles with concanavalin A (Fig. 3c) with no differences in the Pearsons correlation coefficients (Fig. 3d).

**Figure 4:**
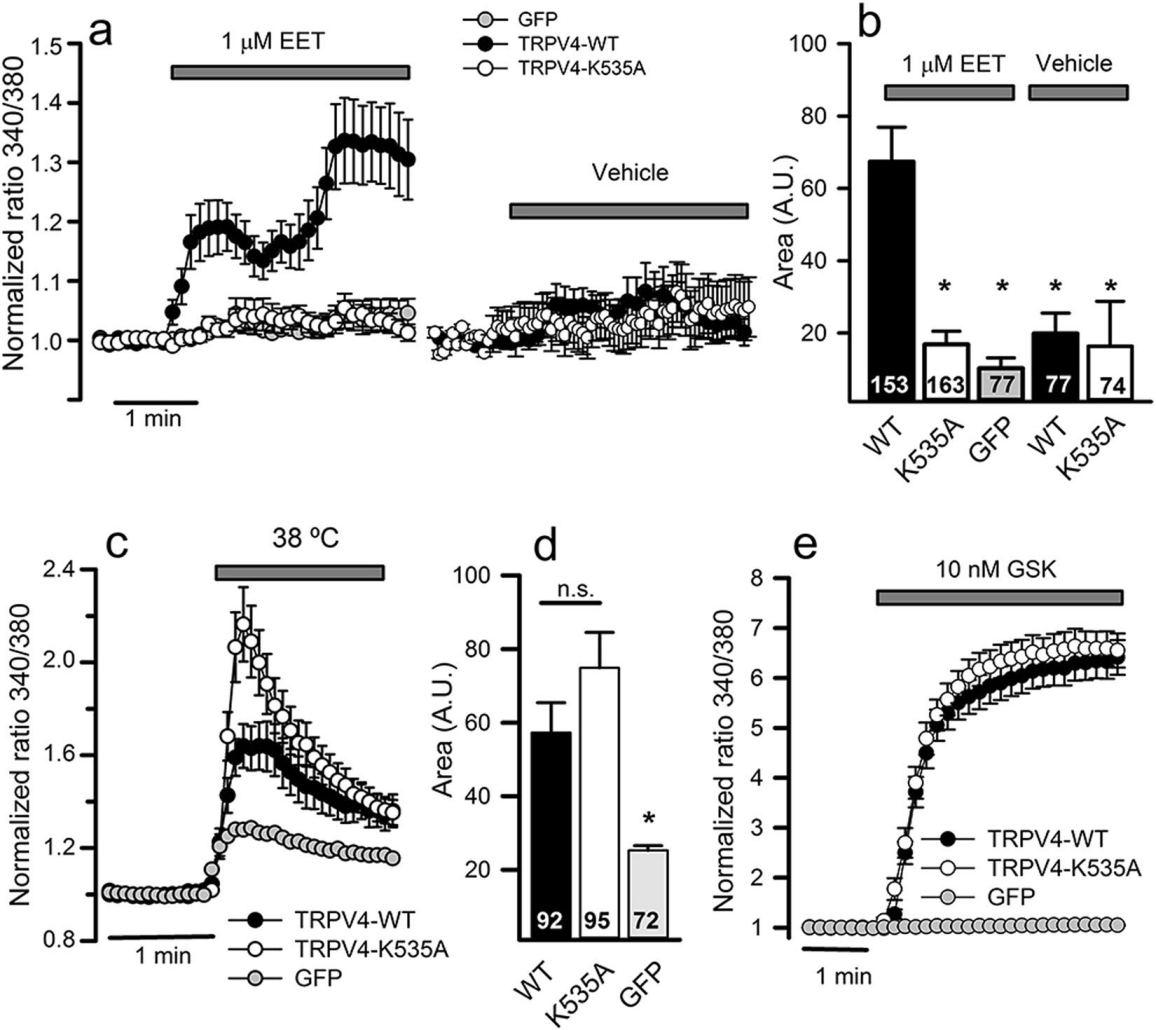
TRPV4-K535A channel loses activation by 5′,6′-EET not by heat or GSK1016790A agonist. (**a**) Changes in intracellular [Ca^2+^] (indicated by normalized fura-2 ratios) in HeLa cells transfected with GFP, TRPV4-WT or TRPV4-K535A cDNAs, after perfusion with 1 µM 5′,6′-EET or vehicle (as indicated). Traces are means ± SEM of 74–163 cells measured in 4–8 independent experiments. (**b**) Average [Ca^2+^]_i_ increases (area under the curve) from traces shown in a. Numbers inside the bars indicate the number of cells analyzed. **(c)** Changes in intracellular [Ca^2+^] in response to warm solution (38 °C). (**d)** Average [Ca^2+^]_i_ increases (area under the curve) from traces shown in c. (**e**) Changes in intracellular [Ca^2+^] in response to 10 nM GSK1016790A (GSK). Traces are means of 93–109 cells measured in 4–6 independent experiments. *P < 0.05 or not significant (n.s.) when compared with cells expressing TRPV4 WT channels (Kruskal-Wallis followed by Dunn *post hoc* test).

By using intracellular Ca^2+^ imaging with Fura-2, we evaluated the sensitivity to 5′,6′-EET (1 μM), heat (38 °C) and the synthetic agonist GSK1016790A (10 nM) of TRPV4-WT, TRPV4-K535A and TRPV4-K535Q channels heterologously expressed in HeLa cells. Addition of 1 μM 5′,6′-EET evoked a significant [Ca^2+^]_i_ response in cells transiently transfected with TRPV4-WT but not in cells expressing TRPV4-K535A (Fig. 4a,b **)** or TRPV4-K535Q (Supplementary Fig. 8a) mutant channels, whose response to 5′,6′-EET was indistinguishable from that obtained in control GFP-transfected cells or in TRPV4-transfected cells treated with vehicle (Fig. 4a,b and Supplementary Fig. 8a). In contrast, K535A or K535Q mutations had no significant effect on the TRPV4-mediated [Ca^2+^]_i_ responses to heat, GSK1016790A (Fig. 4c–e and Supplementary Fig. 8c-e) or 4α-PDD (Supplementary Fig. 9). Besides, the TRPV4-mediated increase of [Ca^2+^]_i_ in response to AA, the EETs precursor, was also lost in cells transfected with mutant channels (Supplementary Fig. 8b).

**Figure 5:**
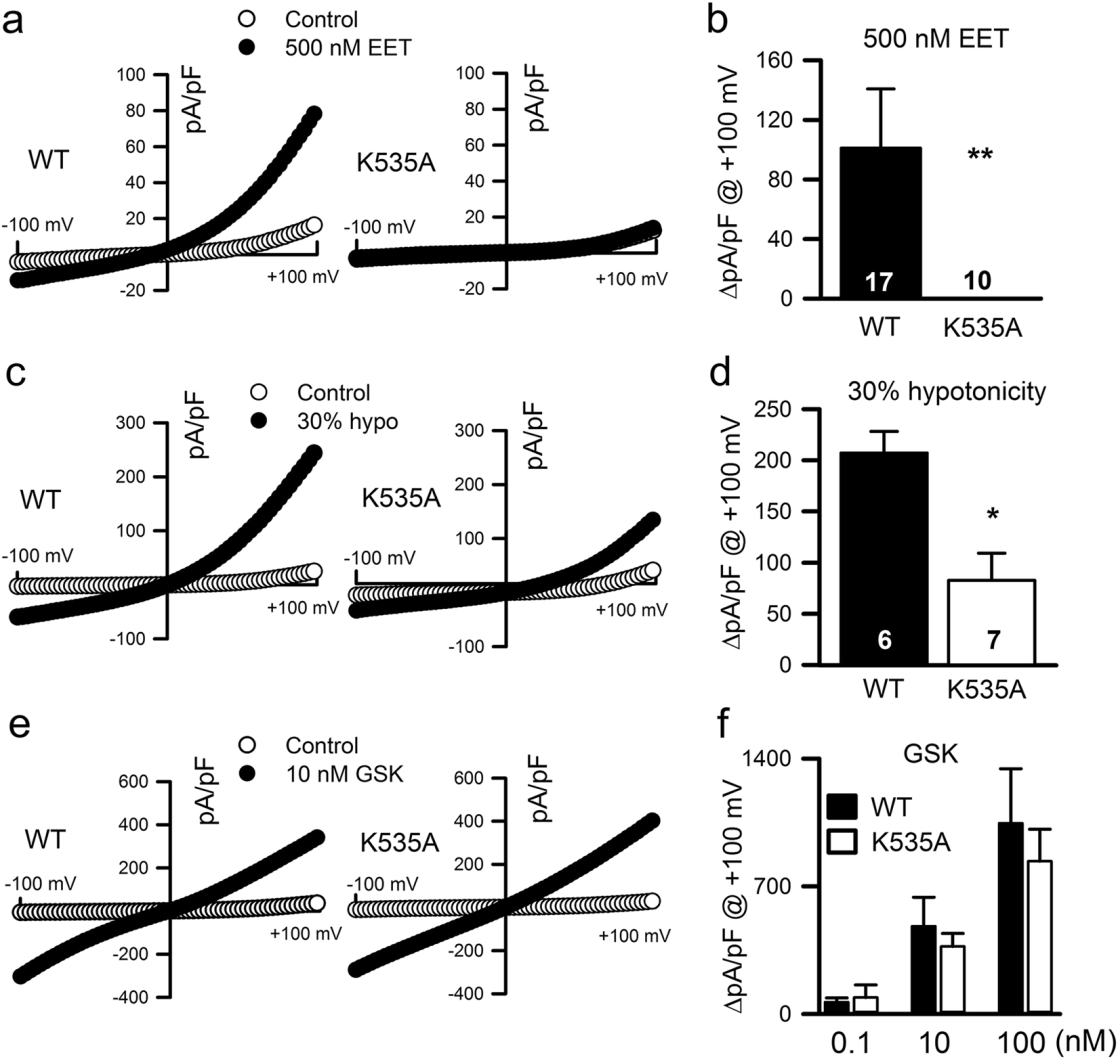
Effect of K535A substitution on the activation of TRPV4 whole-cell currents. Representative ramp current desnsity-voltage relations of whole-cell cationic currents recorded from HEK293 cells transfected with either TRPV4 WT or TRPV4 K535A cDNAs before (control) and after dialysis with 500 nM 5′,6′-EET **(a)**, exposition to a 30% hypotonic shock **(c)** or to 10 nM GSK1016790A **(e)**. **(b**,**d**,**f)** Average TRPV4 current density (at + 100 mV) increases in response to the above indicated stimuli. Data are expressed as the mean ± SEM, and the number of cells recorded is shown for each experimental condition. **P < 0.01, *P < 0.05 K535A *versus* WT. P values in panel b = 0.007, panel d = 0.002 and > 0.05 at al concentrations in panel f (One-tailed Student′s unpaired *t* test).

Consistent with the calcium imaging measurements, the use of electrophysiological techniques demonstrated that 5′,6′-EET (Fig. 5a,b **)** and AA (Supplementary Fig. 10) only increased channel activity in HEK293 cells transiently transfected with TRPV4-WT but not in cells expressing TRPV4-K535A or TRPV4-K535Q mutant channels. Moreover, supporting the idea that TRPV4 activation by osmotic stress depends mostly on phospholipase A_2_ (PLA_2_) activation, AA production and its subsequent metabolism to EET 33, mutation K535A strongly reduced TRPV4 whole-cell currents elicited by a 30% hypotonic shock (Fig. 5c,d). TRPV4-mediated whole-cell currents elicited by GSK1016790A (0.1, 10 and 100 nM) were unaffected by mutation K535A (Fig. 5e,f).

Two more residues (F549 and Q550), in close proximity to K535, that in the 120 ns MD trajectory also showed frequent interactions with 5′,6′-EET ( > 60% of the time) were mutated (F549A and Q550A) and tested for their response to AA, heat and GSK1016790A (Supplementary Fig. 11a-c). While TRPV4-Q550A seems to be a dead channel, unresponsive to all three stimuli, TRPV4-F549A showed reduced response to GSK1016790A without significant changes in the response to AA and heat. We also mutated residue D743, which establishes a salt bridge with K535. TRPV4-D743Q channel showed a reduced response to GSK1016790A but normal activity when challenged with 1 μM 5′,6′-EET (Supplementary Fig. 11d-e).

**Figure 6:**
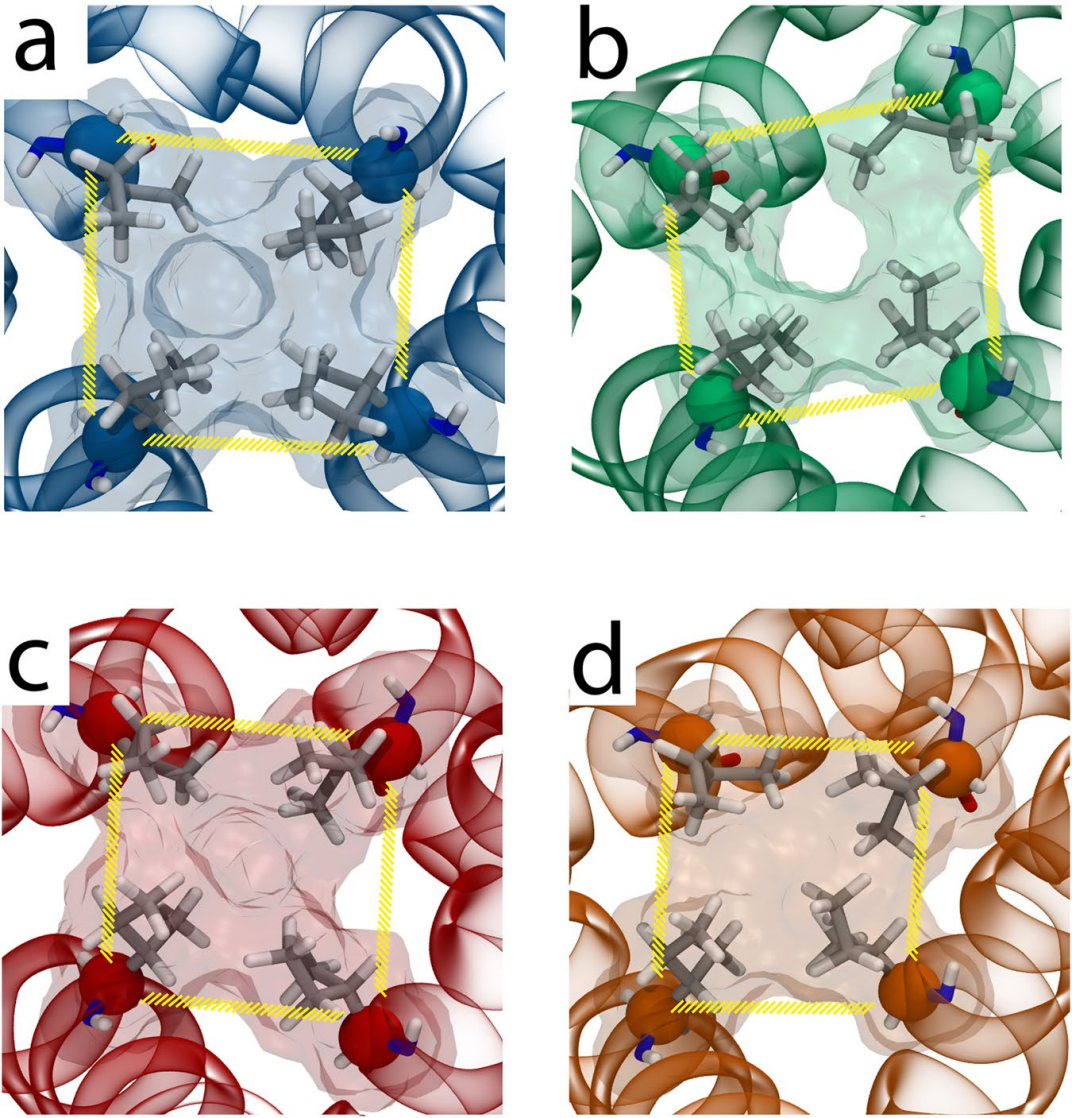
K535A substitution prevents the 5′,6′-EET-induced change in TRPV4 pore dimension. Pore area delimited by the square formed by the four Cα of I715 residues (located at the narrowest zone of TRPV4 pore). (**a**) TRPV4-WT; (**b**) TRPV4-WT + 5′,6′-EET; (**c**) TRPV4-K535A; (**d**) TRPV4-K535A + 5′,6′-EET.

**Figure 7:**
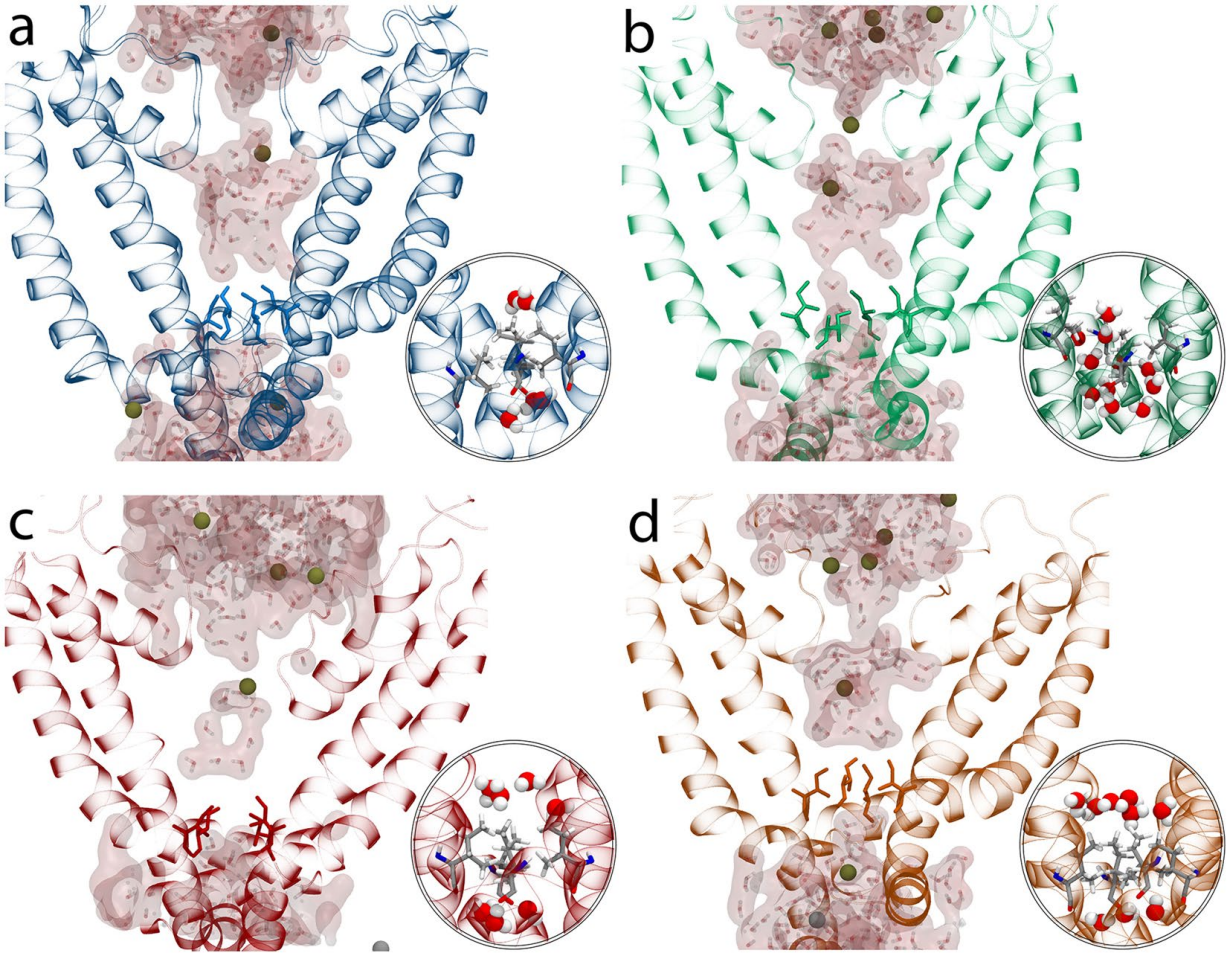
K535A substitution abolishes the 5′,6′-EET-induced water flux through TRPV4 pore. Water presence around I715 residue in TRPV4-WT **(a)**, TRPV4-WT + EET **(b)**, TRPV4-K535A **(c)**, and TRPV4-K535A + EET **(d)** systems. Insets: Water molecules crossing the hydrophobic seal formed by I715 are observed exclusively in the TRPV4-WT + EET system.

Finally, we assessed whether changes in the size of the TRPV4 channel pore caused by 5′,6′-EET binding could be observed during MD simulation and may be affected by the K535A mutation. For that purpose, using both TRPV4 WT and K535A systems in the absence and presence of 5′,6′-EET, we measured the area determined by a square formed by the four α-carbons of I715 residues (Fig. 6). These residues are located at the narrowest zone of TRPV4 pore and are homologous to I679 residues that in the TRPV1 structure form a hydrophobic seal (lower gate) that expands in the presence of channel agonists^48, 49^. For all four systems studied, the area started at the same value (~55Å^2^), and only for the TRPV4 WT system in the presence of 5′,6′-EET (Fig. 6b) the area increased from 55.5 ± 2.9 Å^2^ up to 63.8 ± 4.2 Å^2^ (mean ± S.D.: P = 0.0001 as determined by one way ANOVA followed by a Bonferroni post hoc test). Besides, the opening of the pore in the presence of 5′,6′-EET was unrelated to changes in the distance between Cα of residues W733 and R594 (WT: 8.0 ± 0.56 in the absence and 7.8 ± 0.59 in the presence of 5′,6′-EET, mean ± S.D.; P > 0.05) or L596 (WT: 5.9 ± 0.5 in the absence and 5.6 ± 0.3 in the presence of 5′,6′-EET, mean ± S.D.; P > 0.05), which has been recently linked to alterations in channel activity^50^. In the TRPV4-K535A system, the presence of 5′,6′-EET did not increase the area (56.4 ± 4.4 Å^2^ versus 53 ± 2.6 Å^2^, in the absence and presence of 5′,6′-EET, respectively) (Fig. 6c,d). Accordingly, only in the pore of the TRPV4-WT + EET system (Fig. 7) was possible to observe water molecules crossing the hydrophobic seal formed by I715. Together, our results support the view that 5′,6′-EET binds TRPV4 and its sufficient to gate channel opening.

## Discussion

TRPV4 channel gating by EETs is physiologically relevant in the context of vasodilatation^14, 21, 37, 38, 51–54^. In the brain, glial cells sense high Na^+^ levels to produce EETs that in turn activate neuronal TRPV4 to trigger water intake 41 and the homeostatic control of body fluid osmolarity. Besides, TRPV4 mechano/osmosensitivity depends on PLA_2_ activation and the subsequent production of the P450-mediated AA metabolites, EETs^8, 33, 34^. However, the lack of evidence for a direct interaction of EET with TRPV4 and claims of EET-independent activation of TRPV4 by AA^55^ and mechanical stimulation^56, 57^ have cast doubts on the validity of this mechanism.

Our study presents two major findings: 1) The identification of an EET-binding pocket at the TRPV4 channel that specifically participates in the channel activation by 5′,6′-EET, AA and hypotonic cell swelling, thereby supporting the view that all these stimuli shared a common final structural target within the TRPV4 channel; although we do not discard other modes of swelling-induced TRPV4 activation as the response of the TRPV4-K535A channel to hypotonic stimulation is not completely abrogated; and 2) The first structural insight into the gating of TRPV4 by a natural (lipidic) agonist (5′,6′-EET).

The EET binding site identified involves residues from S2-S3 linker (K535, F549 and Q550), S4 (Y591) and S4-S5 linker (R594). Remarkably, all these amino acids are conserved throughout evolution and, among these residues, K535 plays a crucial role in 5′,6′-EET binding to TRPV4, as the mutant TRPV4-K535A channel loses the capability of binding 5′,6′-EET and the response to 5′,6′-EET, AA and hypotonicity, without affecting TRPV4 gating by temperature or the TRPV4 agonists GSK1016790A and 4α-PDD. Consistent with a direct and sufficient interaction of 5′,6′-EET with TRPV4 to activate the channel, molecular dynamics simulation showed that 5′,6′-EET triggers pore opening of the TRPV4-WT but not the TRPV4-K535A channel.

To date, the structures of two members of the vanilloid family (TRPV1 and TRPV2)^48, 49, 58, 59^ have been resolved with high, near-atomic resolution, and many of their features are considered to be shared by TRPV4^46, 50, 60^. These structural studies have identified two putative lipid binding sites in rabbit TRPV2. One is located near the C-terminal portion of S4 (from N509 to R514, a region highly conserved between human TRPV1, TRPV2 and TRPV4 (Figure supplement 12)), the S4-S5 linker and the S6 of an adjacent subunit^58^. A similar lipid binding site has been reported for rat TRPV1, which overlaps with the binding-pocket for capsaicin and other vanilloid ligands^59^. In addition, comparison of TRPV1 structural data obtained in the absence (closed state) and presence of resiniferatoxin (a capsaicin analog) suggest that channel activation by vanilloids entails the displacement of the resident lipid, the stabilization of vanilloid binding by Y511 (at S3) and the vanilloid-coordinated interaction between two residues (R557 and E570) to pull the S4-S5 linker and facilitate opening of the lower gate. This model is supported by functional studies showing that R557 and E570 mutations impairs the allosteric coupling between the voltage-, temperature- and capsaicin-dependent TRPV1 activation mechanisms, which is partially restored by the charge-swapping double mutation R557E/E570R^61^. It is worth mentioning that R557 is a conserved residue among human TRPV1, TRPV2 (R515) and TRPV4 (R594). Moreover, R594 (along with the S4 residue Y591, which we have identified in the EET-binding pocket) has been reported to play a general role in the transduction of chemical stimuli into TRPV4 gating 47, an observation we also reproduce here (Supplementary Fig. 6). Consistent with this important function, TRPV4 mutations of R594 that promote TRPV4 basal activity have clinical consequences in the context of skeletal dysplasias^62^.

The other reported TRPV2 lipid binding site, has been placed in the crevice formed by the S1-S4 helical bundle^58^, a location that also accommodates lipids in the TRPV1 structure^48, 59^. The TRPV4 EET-binding site we now report also relates to this crevice formed by segments S1 through S4. Among the S1-S4 amino acids we have identified within a distance of 3.5 Å of 5′,6′-EET, Y591 is identical in both TRPV1 and TRPV2 channels (Supplementary Fig. 12). Of particular relevance, residue K535, with a key role in the EET binding-induced TRPV4 gating, corresponds to Q498 in TRPV1 (Supplementary Fig. 12), a change (K to Q) that abrogates TRPV4 response to 5′,6′-EET, and that might account for the EET-insensitivity of TRPV1.

The S1-S4 TRPV4 cleft containing the EET-binding pocket is placed above the TRP box domain, and according to TRPV1 and TRPV2 structural data it is a hot spot for controlling channel gating. Actually, the interaction of the agonist 4α-PDD with TRPV4 has also been positioned at the pocket between S3 and S4 segments and of the three amino acids (Y556, L584 and W586) 47 involved in such interaction, Y556 is also one of the residues frequently interacting with 5′,6′-EET in the TRPV4-WT MD simulation. How EET and 4α-PDD interaction with TRPV4 leads to channel opening remains to be elucidated. Previous TRPV4 MD simulations showed that W733 (TRP box) usually interacts with L596 (but also with R594, in the S4-S5 linker) and that changes in this distance will disrupt the latch that keeps the channel close promoting its aperture^50^. Considering that R594 is one of the key residues for EET binding, we speculated that agonist-induced conformational changes might increase the distance between W733 (sited at the TRP box) and L596 or R594 (both positioned at the S4-S5 linker) to open the channel. However, we could not detect changes in this distance in the presence of 5′,6′-EET in any of the MD systems analyzed.

In conclusion, we show here, for the first time, that direct 5′,6′-EET binding to TRPV4 gates the channel. We have located the EET-binding pocket in close relationship with segments S2-S3 linker through S4, into the S1-S4 crevice above the TRP box, with partial overlapping with the predicted binding site of 4α-PDD between S3 and S4. Moreover, the fact that mutant TRPV4-K535A channel loses EET binding and EET-, AA- and hypotonicity-induced activation without affecting GSK, 4α-PDD and temperature activation suggested a common structural motif within TRPV4 that is targeted by the swelling-PLA_2_-AA-EET pathway.

## Methods

### Cells and transfections

For electrophysiological or calcium imaging experiments HeLa and HEK293 cells were transiently transfected with human TRPV4 wild-type/mutant cDNAs cloned in the pcDNA3.1 vector as previously described^34^.

### Confocal microscopy

Cells transiently transfected with human TRPV4 wild-type or mutants (in a pcDNA3.1 vector were stained with 100 μg/ml of the plasma membrane marker concanavalin A tetramethylrhodamine (Invitrogen) for 20 min on ice and then fixed with 4% paraformaldehyde and probed with a polyclonal affinity-purified anti-human TRPV4 (1:1000)^63^ at room temperature for 1 hour. Digital images were taken and analyzed using a Leica TCS SP2 and the National Institutes of Health Image J software (http://rsb.info.nih.gov/ij/).

### TRPV4 Docking and Molecular Dynamics Simulations

Human TRPV4 and 5′,6′-EET were prepared for docking using the DockPrep tool in UCSF Chimera^64^. The docking calculations were performed with the AutoDock Vina tool (Scripps Research Institute, La Jolla, CA)^65^ included in UCSF Chimera. 10 docking rounds providing 10 docking poses each (total of 100 docking poses) were initially performed using a 100 Å × 100 Å × 50 Å docking box around the cytosol-bilayer interface of TRPV4. Further docking refinement was performed using smaller docking boxes (20 Å × 20 Å × 20 Å) around the defined binding site, to obtain larger binding energy values.

All MD systems analyzed were based on a previously reported TRPV4 MD system^46^. The putative EET location predicted by the docking pose with higher energy (−7.4 kcal/mol), with the grid located at the mass center of residue 400, was used as starting point for subsequent MD simulations. In the presence of 5′,6′-EET, the force field parameters were obtained from ParamChem^66^. Harmonic restraints of 1 kcal/mol/Å^2^ were initially set on the α-carbons of the secondary structure and were diminished by 0.2 kcal/mol/Å^2^ in periods of 1 ns. Subsequently, 120 ns of trajectory were obtained. All calculations were run using NAMD 2.9^67^.

### Microscale Thermophoresis

MST analysis was performed using a NanoTemper Monolith NT.115 instrument, as previously described^68^. Briefly, C-tail tagged TRPV4-WT-GFP-His, TRPV4-K535A-GFP-His, TRPV4-R594A-GFP-His and GFP-His were solubilized from HEK293 cells transfected with pCDNA3-TRPV4-GFP-His or pCDNA3-TRPV4-K535A-GFP-His and treated with a lysis buffer containing: 2% DDM, 300 mM NaCl, 50 mM Tris HCl, pH8.0. Subsequently, proteins of interest were enriched by FPLC using Ni-NTA columns (HisTrap FF Crude, GE Healthcare Life Sciences). The different fractions were separated by an imidazole gradient (0–500 mM): buffer A (0.025% DDM, 150 mM NaCl, 50 mM TrisHCl, pH8.0) + buffer B (0.025% DDM, 150 mM NaCl, 50 mM Tris-HCl, 500 mM imidazole). A concentration of 20 nM TRPV4 or TRPV4-K535A was incubated in the dark with different concentrations of 5′,6′-EET (Santa Cruz Biotechnology, USA) in Buffer A (0.025% DDM, 150 mM NaCl, 50 mM Tris HCl, pH8.0) containing 2% ethanol as vehicle. The samples were loaded into standard glass capillaries (Monolith NT.115 Capillaries) and thermophoresis analysis was performed (LED 40%, IR laser 80%). Dissociation constants were calculated using the NanoTemper 1.2.206 analysis program.

### Ratiometric Ca^2+^ Measurements, Electrophysiological Recordings and Solutions

Cytosolic Ca^2+^ signals, relative to the fluorescence ratio (340/380) measured prior to cell stimulation, were obtained from transfected HeLa cells loaded with 4.5 μM fura-2 AM (Invitrogen) as previously described^34^. Isotonic bath solutions used for Ca^2+^ imaging experiments contained 140 mM NaCl, 2.5 mM KCl, 1.2 mM CaCl_2_, 0.5 mM MgCl_2_, 5 mM glucose, and 10 mM HEPES (pH 7.3, adjusted with Tris).

For patch-clamp whole-cell recordings on transfected HEK293 cells, bath solutions contained 100 mM NaCl, 1 mM MgCl_2_, 6 mM CsCl, 10 mM HEPES, 1 mM EGTA, and 5 mM glucose (pH 7.3, adjusted with Tris). Osmolarity was adjusted to 310 mOsm/L adding D-mannitol (isotonic solution) and 30% hypotonic solution (220 mOsm/L) were obtained by omitting D-mannitol. Borosilicate glass electrodes (2–3 MΩ) were filled with a solution contained 20 mM CsCl_2_, 100 mM CsAcetate, 1 mM MgCl_2_, 0.1 mM EGTA, 10 mM HEPES, 4 mM Na_2_ATP, and 0.1 mM NaGTP; 300 mOsm/L (pH 7.25, adjusted with CsOH). When required, 500 nM 5,6-EET was added to this intrapipette solution. Home-made local perfusion^69^ was used to deliver TRPV4 agonist (GSK1016790A, Tocris Bioscience, UK), antagonist (HC067047, Tocris Bioscience, UK) and solutions of different osmolality.

TRPV4 activity was assessed with a 400 ms ramp from −100 mV to +100 mV using a D-6100 Darmstadt (List Medical, Germany) amplifier and pClamp 8 software (Molecular Devices, USA). The ramp protocol was applied every 5 seconds in the absence (control) or presence of TRPV4 stimuli. TRPV4-specific responses were always validated using the TRPV4 inhibitor HC067047 (10 µM) in presence of the selected activating stimuli. Cell-attached recordings were obtained from patches in EGFP-positive HEK293 cells 2 days after transfection with cDNA encoding wild-type (WT), K535A or K535Q mutant TRPV4 channels, clamped at +80 mV using a gap-free protocol and an Axopatch 200 A (Molecular Devices, U.S.A.) or an EPC10-USB patch-clamp amplifier (HEKA, Germany). Borosilicate glass patch pipettes had a tip resistance of 4–5 MΩ and were filled with a solution containing 140 mM NaCl, 1 mM MgCl_2_, 5 mM CsCl, 1 mM EGTA, 5 mM Glucose and 10 mM HEPES (310 mOsm/L, pH 7.35). The bath solution contained 140 mM KCl (to zero the membrane potential), 1 mM MgCl_2_, 5 mM Glucose, and 10 mM HEPES (300 mOsm/L, pH 7.4 adjusted with Tris).

The pClamp10.5, PatchMaster, FitMaster, Fetchan and pStat softwares were employed for voltage-clamp, data acquisition, and subsequent analysis of all electrophysiological data. Whole-cell and cell-attached currents were recorded at 10 kHz and low-pass-filtered at 1 kHz. Cell-attached activity of WT and mutants TRPV4 channels was assessed as the NP_O_ (number of channels x single channel open probability) from 15 seconds continuous recordings at +80 mV obtained in the presence of extracellular arachidonic acid or the corresponding vehicle (DMSO, 1:500 dilution), which were added to the intrapipette solution.

Electrophysiological and ratiometric Ca^2+^ recordings were obtained at room temperature (24 °C), unless otherwise indicated.

All chemicals were obtained from Sigma-Aldrich unless otherwise indicated.

### Statistical Analysis

Data are expressed as mean ± SEM (unless otherwise indicated). Statistical analysis was assessed with Student′s unpaired *t* test, Mann-Whitney U-test, one way analysis of variance (ANOVA) followed by Bonferroni *post hoc* test, or Kruskal-Wallis test followed by Dunn *post hoc* test, as appropriate. Differences were considered significant if P < 0.05.

### Data Availability

All data generated or analyzed during this study are included in this published article (and its Supplementary Information files).

## Electronic supplementary material

Supplementary Info

video 1

video 2
