# Supplementary material for: Structural determinants of 5′,6′-epoxyeicosatrienoic acid binding to and activation of TRPV4 channel
Source: Sci Rep. 2017 Sep 5;7:10522. doi: 10.1038/s41598-017-11274-1 (PMC5585255; doi:10.1038/s41598-017-11274-1)
Supplement: Supplementary file 1 — Supplementary Info [file 41598_2017_11274_MOESM1_ESM.pdf]

Structural determinants of 5’,6’-epoxyeicosatrienoic acid binding to and activation of TRPV4 channel

Alejandro Berna-Erro[a,1], Mercè Izquierdo-Serra[a,1], Romina V. Sepúlveda[b], Fanny Rubio-Moscardo[a], Pau Doñate-Macián[a], Selma A. Serra[a], Julia Carrillo-García[a], Alex Perálvarez-Marín[c], Fernando González-Nilo[b,d], José M. Fernández-Fernández [a,2] and Miguel A. Valverde[a,2]

[a]Laboratory of Molecular Physiology, Dept. of Experimental and Health Sciences, Universitat Pompeu Fabra, Barcelona, Spain.

[b]Universidad Andrés Bello, Center for Bioinformatics and Integrative Biology, Facultad de Ciencias Biológicas, Av. República 239, Santiago, Chile.

[c]Unitat de Biofísica, Centre d’Estudis en Biofísica, Departament de Bioquímica i de Biologia Molecular, Universitat Autònoma de Barcelona, 08193 Bellaterra, Spain

[d]Centro Interdisciplinario de Neurociencia de Valparaíso, Facultad de Ciencias, Universidad de Valparaíso, Valparaíso 2366103, Chile.

[1]Contributed equally to this work

[2]Corresponding authors: Prof. Miguel A. Valverde and Dr. José M. Fernandez-Fernández Laboratory of Molecular Physiology, Universitat Pompeu Fabra, C/ Dr. Aiguader 88, Barcelona 08003, Spain. Phone: 34 93 3160853, Fax: 34 93 3160901. Email: miguel.valverde@upf.edu ; jmanuel.fernandez@upf.edu

SUPPLEMENTARY INFORMATION

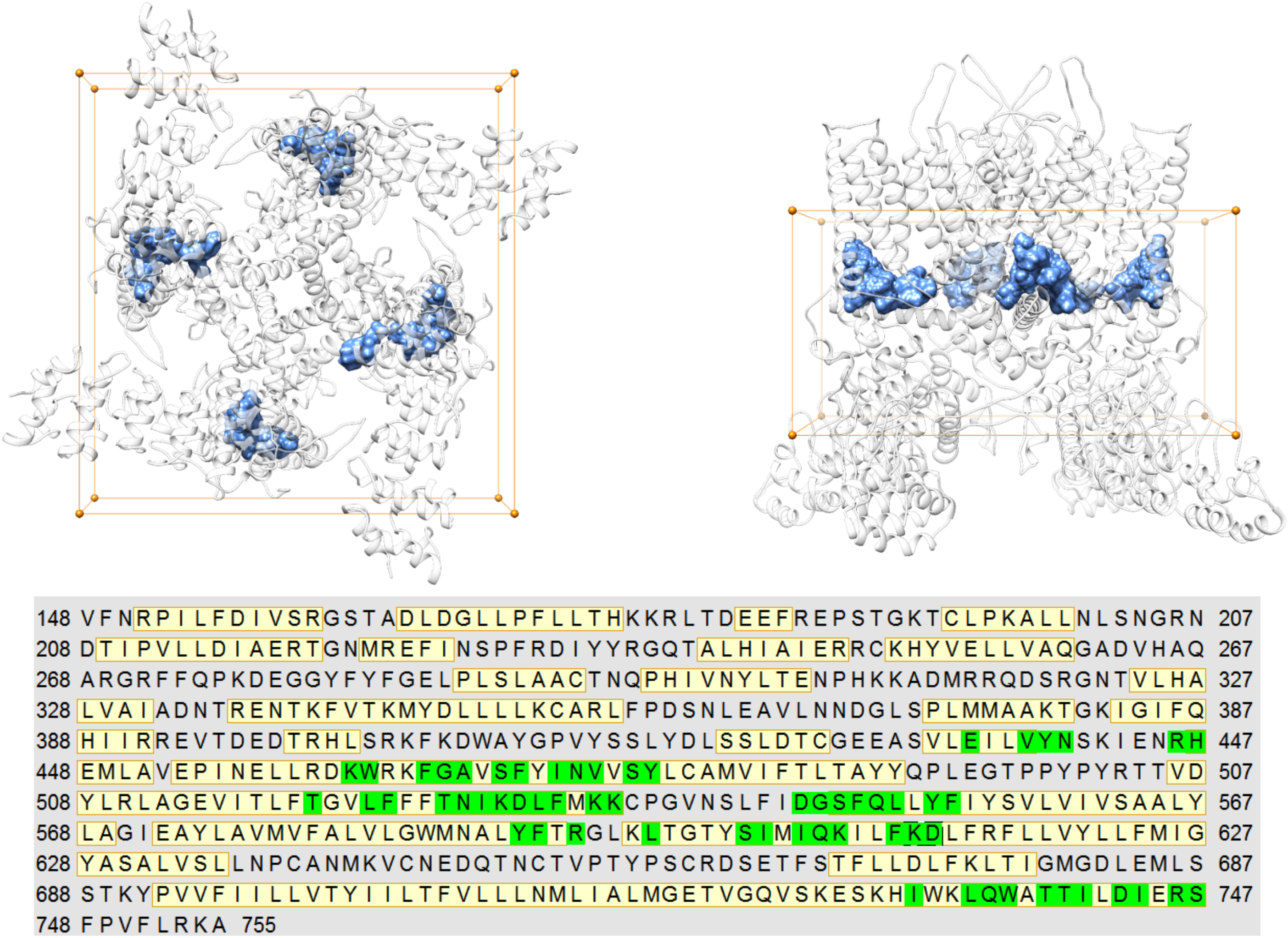

Figure supplement 1. 5’,6’-EET blind docking. Top, TRPV4 bottom and side views (white ribbons) with 100 5’,6’-EET docking poses (light blue surface) defining the putative binding site. The docking box of 100Å x 100 Å x 50 Å is indicated by the orange line box. **Bottom,** TRPV4 residues (green highlight) within a 5 Å distance of the 5,6-EET docking poses.

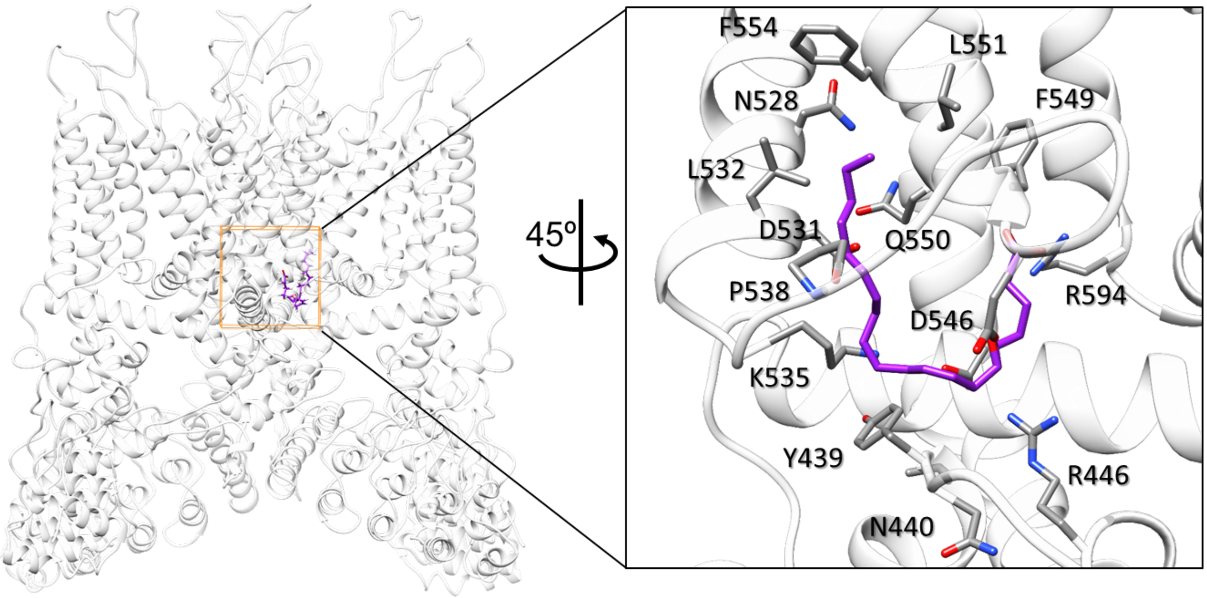

Figure supplement 2. 5’,6’-EET refined docking. TRPV4 side view (white ribbons) with the 5’,6’-EET highest energy docking solution (in purple) defining the putative binding site. The docking box of 20Å x 20 Å x 20 Å is indicated by the orange line box. The zoomed 5’,6’-EET-binding site highlights the TRPV4 residues within a 3.5 Å distance of 5’,6’-EET.

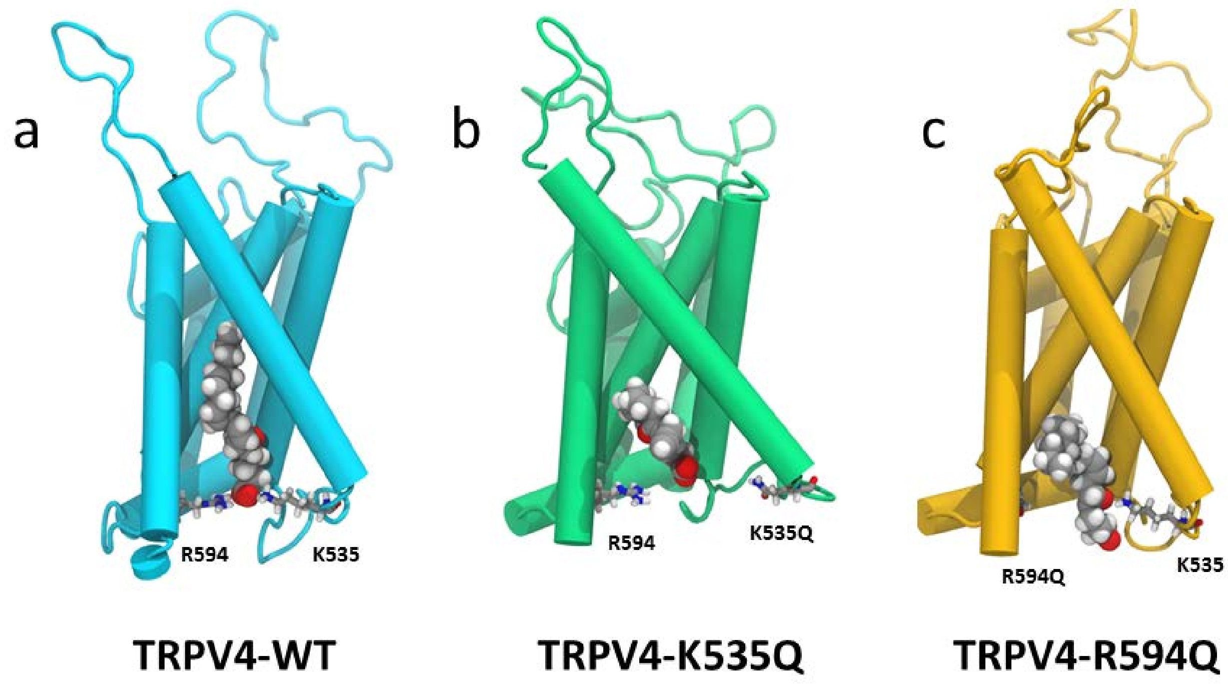

Figure supplement 3. Atomic-level interactions between 5’,6’-EET and residues K535 and R594 of a single subunit in the TRPV4-WT (**a**), TRPV4-K535Q (**b**) and TRPV4-R594Q (**c**) systems. Note the increased distance between 5’,6’-EET and the mutated residues K535Q and R594Q**.**

S2-S3 linker

```
Human      (Q9HBA0)      530 KDLFMKKCPGVNSLFIDGSFQ 550
Bovin      (E1BCP0)      530 KDLFMKKCPGVNSLFIDGSFQ 550
Mouse      (Q9EPK8)      530 KDLFTKKCPGVNSLFVDGSFQ 550
Rat        (Q9ERZ8)      530 KDLFMKKCPGVNSLFVDGSFQ 550
Chicken    (Q9DFS3)      516 KDLFMKKCPGVNSFFIDGSFQ 536
Alligator  (A0A0H5APX6)  521 KDLFMKKCPGVNSFFIDGSFQ 541
Zebrafish  (Q0PEH1)      522 KDLFLKKCPGVNSIFVDGSFQ 542
                             **** ********:*:*****
```

S3

```
Human      (Q9HBA0)      551 LLYFIYSVLVIVSAALYLAGI 571
Bovin      (E1BCP0)      551 LLYFIYSVLVIVSAALYLAGI 571
Mouse      (Q9EPK8)      551 LLYFIYSVLVVVSAALYLAGI 571
Rat        (Q9ERZ8)      551 LLYFIYSVLVVVSAALYLAGI 571
Chicken    (Q9DFS3)      537 LLYFIYSVLVIVTAGLYLGGV 557
Alligator  (A0A0H5APX6)  542 LLYFIYSVLVIVTAGLYVAGI 562
Zebrafish  (Q0PEH1)      543 LLYFIYSVLVVGSAALYLSGI 563
                             **********: :*.**:.*:
```

S4

```
Human      (Q9HBA0)      573 AYLAVMVFALVLGWMNALYFT 593
Bovin      (E1BCP0)      573 AYLAVMVFALVLGWMNALYFT 593
Mouse      (Q9EPK8)      573 AYLAVMVFALVLGWMNALYFT 593
Rat        (Q9ERZ8)      573 AYLAVMVFALVLGWMNALYFT 593
Chicken    (Q9DFS3)      559 AYLAVMVFALVLGWMNALYFT 579
Alligator  (A0A0H5APX6)  564 AYLAVMVFALVLGWMNALYFT 584
Zebrafish  (Q0PEH1)      565 AYVSVMVFALTLGGMNPLYFT 585
                             **::******.** ** ****
```

S4-S5 linker

```
Human      (Q9HBA0)      594 RGLKLTGTYSIMIQKILFKDLFR 616
Bovin      (E1BCP0)      594 RGLKLTGTYSIMIQKILFKDLFR 616
Mouse      (Q9EPK8)      594 RGLKLTGTYSIMIQKILFKDLFR 616
Rat        (Q9ERZ8)      594 RGLKLTGTYSIMIQKILFKDLFR 616
Chicken    (Q9DFS3)      580 RGLKLTGTYSIMIQKILFKDLFR 602
Alligator  (A0A0H5APX6)  585 RGLKLTGTYSIMIQKILFKDLFR 607
Zebrafish  (Q0PEH1)      586 RGLKLTGTYSIMIQKILIKDLFR 608
                             *****************:*****
```

Figure supplement 4. Evolutionary conservation of TRPV4 regions relevant to EET binding. Sequence alignment of S2-S3 linker, S3, S4 and S4-S5 linker of TRPV4 from different species. Residues within a distance of 3.5 Å of EET (according to MD simulation) are shown in purple. Closed arrows indicate identical aa and open arrows conserved or semi-conserved aa. "*" identical residues; ":" conserved substitutions (same amino acid group); "." semi-conserved substitution (similar shapes).

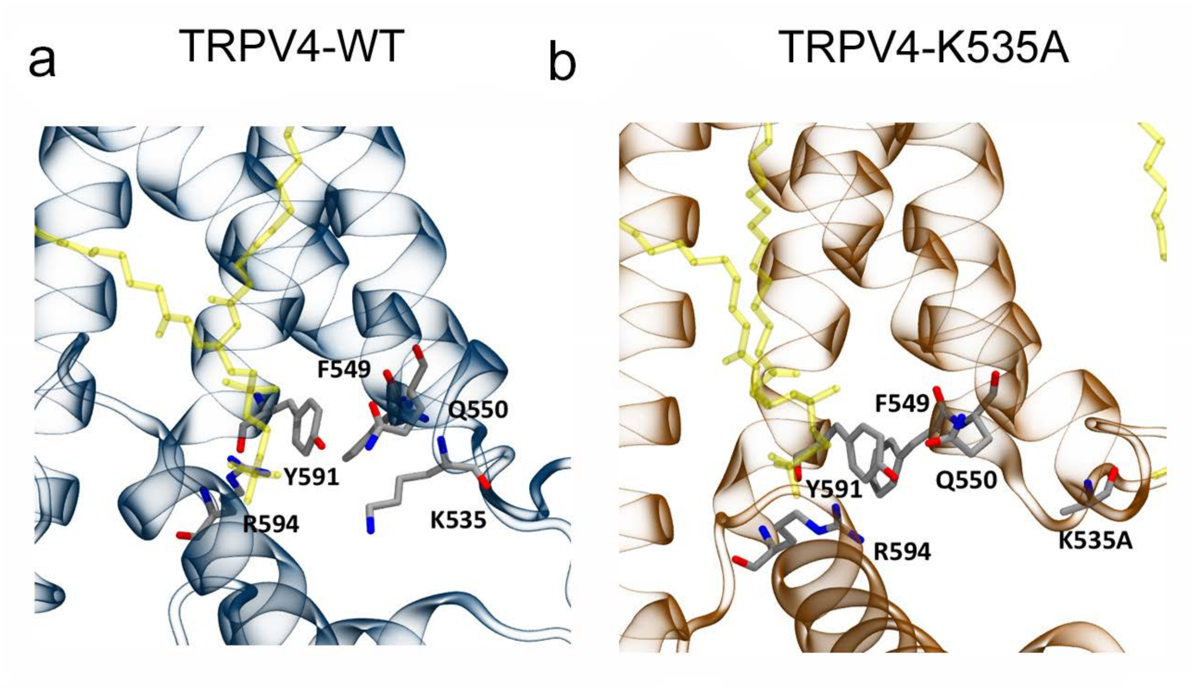

Supplementary Figure. 5. MD simulations in the absence of 5’,6’-EET. Images of the predicted EET-binding site in TRPV4-WT (a) and TRPV4 K535A (b) in the absence of 5’,6’-EET. In yellow are the membrane lipids (POPC) present in the simulation. Note that the predicted EET binding site is empty in both systems.

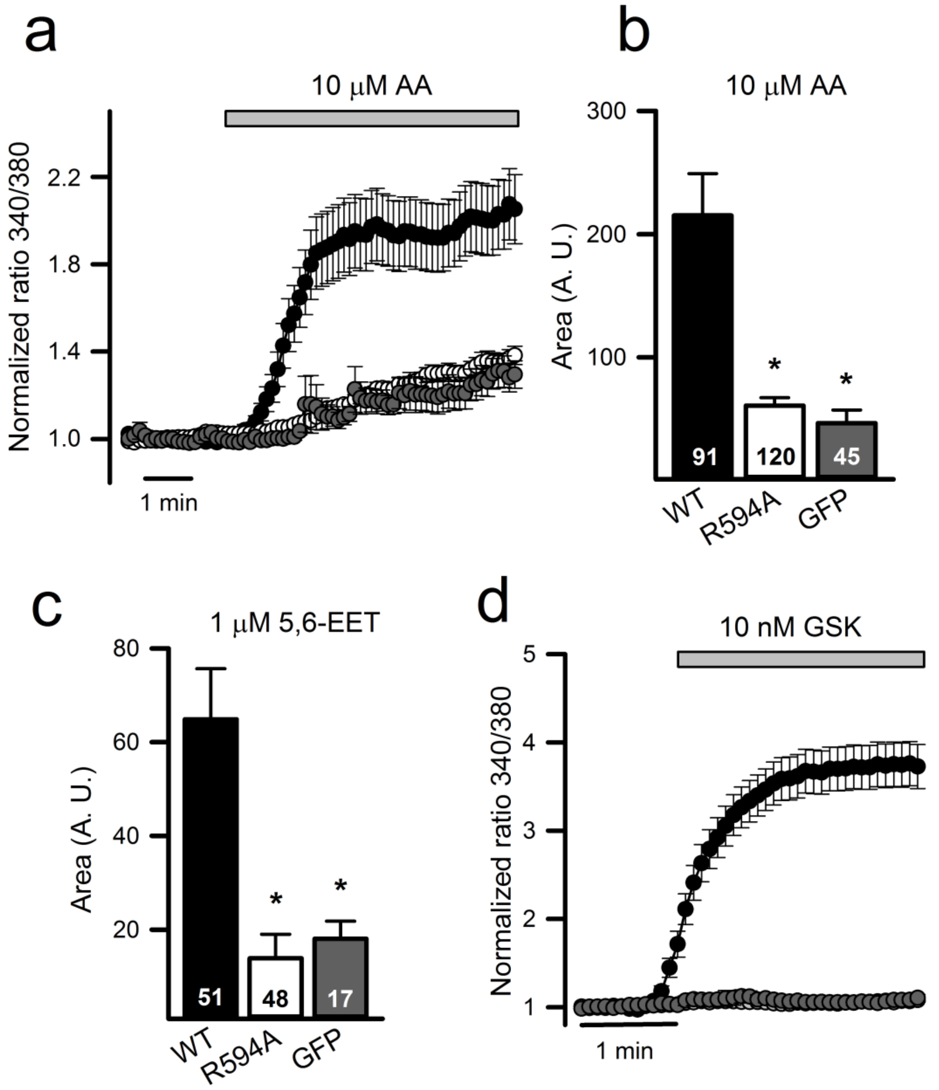

Figure supplement 6. TRPV4-R594A lacks activation by 5',6'-EET, AA and GSK1016790A. Changes in intracellular [Ca^{2+}] (indicated by normalized fura-2 ratios and areas under the curves) in HeLa cells transfected with GFP, TRPV4-WT or TRPV4-R594A cDNAs, after perfusion with 10 µM AA (**a,b**), 1 µM 5,6-EET (**c**) and 10 nM GSK1016790A (**d**). Numbers inside the bars indicate the number of cells analyzed. Means ± S.E.M. *P<0.05 when compared with cells expressing TRPV4-WT channels (Kruskal-Wallis One Way Analysis of Variance **f**ollowed by Dunn's method of multiple comparisons).

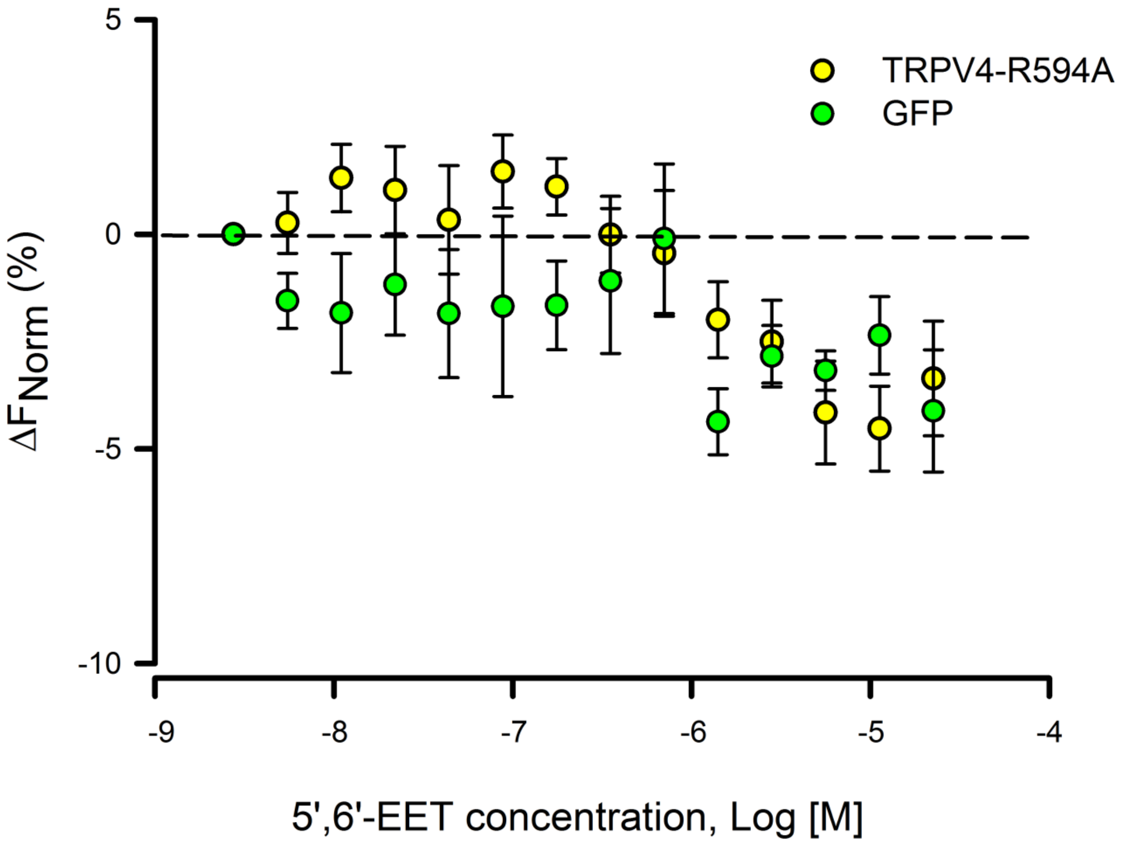

Figure Supplement 7. Thermophoretic analysis of the TRPV4-EET interaction.
Average changes in normalized fluorescence obtained for GFP-labeled TRPV4-R594A (n=5) or GFP (n=5) plotted against 5’,6’-EET concentration.

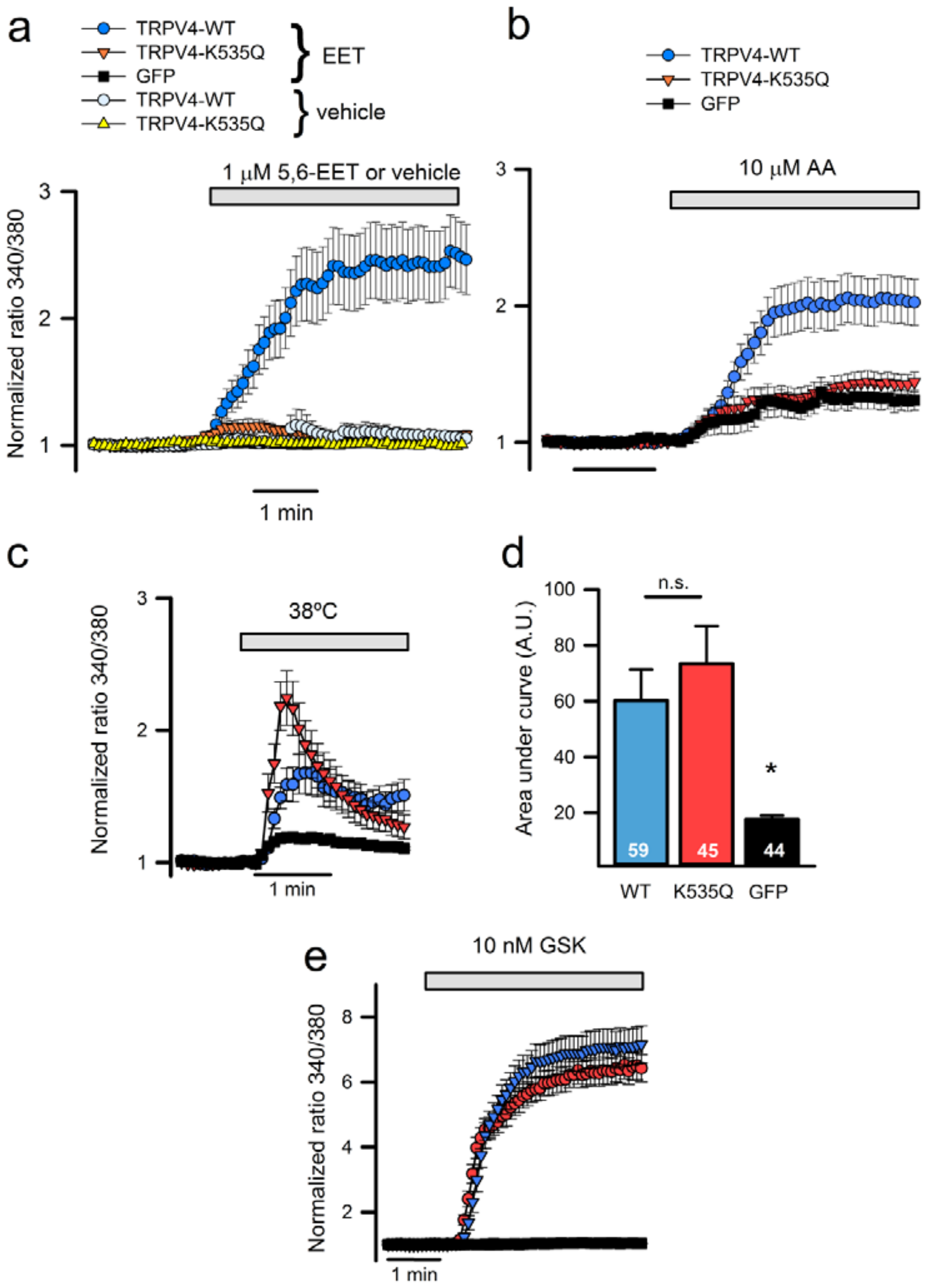

Figure supplement 8. K535Q substitution abolishes TRPV4 activation by 5',6'-EET and AA, but not by heat or GSK1016790A agonist. Intracellular Ca^{2+} changes (indicated by fura-2 ratios) in HeLa cells transfected with GFP (n=50), TRPV4-WT (n=77) or TRPV4-K535Q (n=74) cDNAs, after perfusion with 1 µM 5,6-EET or vehicle (WT, n=51; K535Q, n=20) **(a)**, 10 µM AA (GFP (n=66), TRPV4-WT (n=90) or TRPV4-K535Q (n=90)) (**b**), warm solution (38 ºC) **(c-d)** and 10 nM GSK1016790A (n= 44-59) (**e**). Means ± S.E.M. *P<0.05 or not significant (n.s.) when compared with cells expressing TRPV4 WT channels (Kruskal-Wallis One Way Analysis of Variance **f**ollowed by Dunn's method of multiple comparisons).

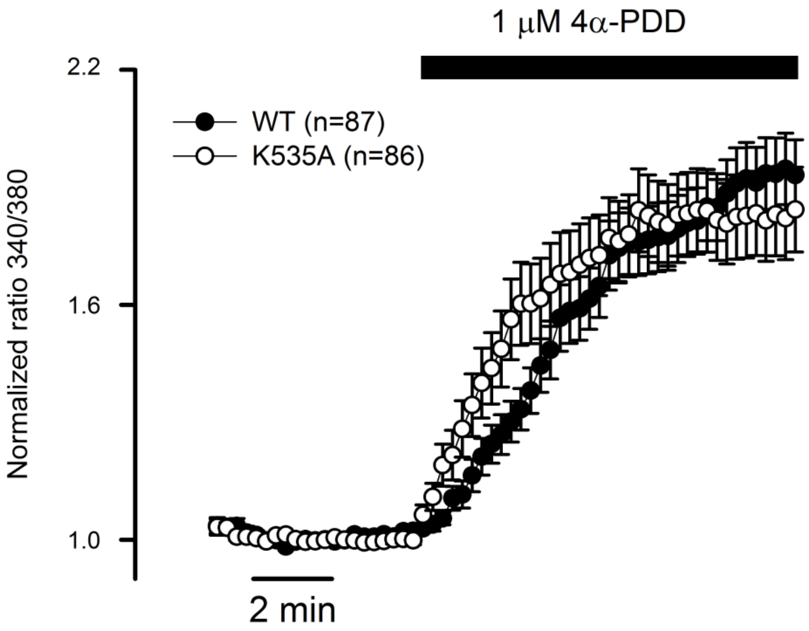

Figure supplement 9. K535A substitution does not abolish TRPV4 activation by 4α-PDD. (A), Fura-2 ratios obtained in HeLa cells transfected with TRPV4-WT (n=87) or TRPV4-K535A (n=86) cDNAs, after perfusion with 10 μM 4α-PDD. Traces showing means ± SEM..

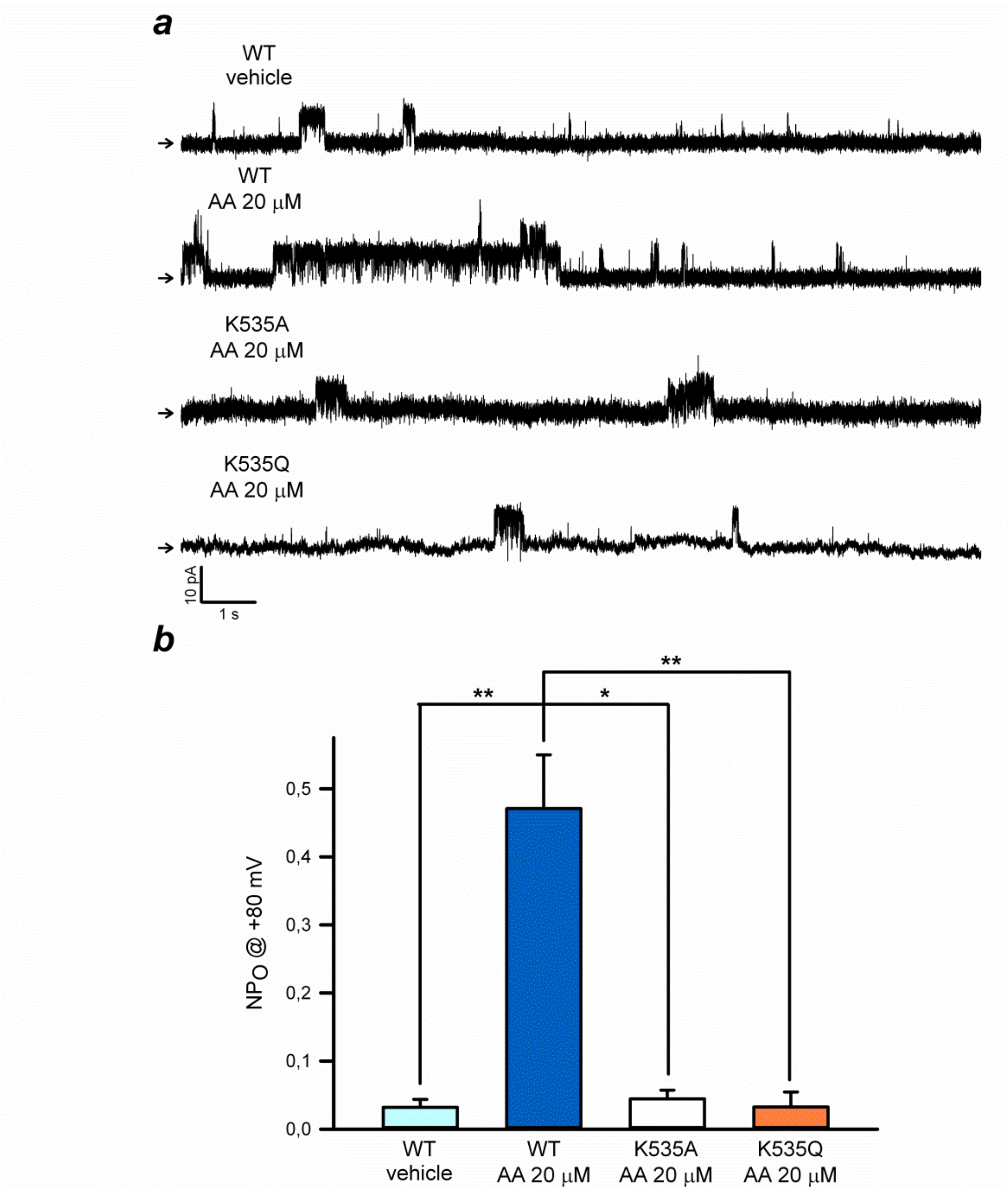

Figure supplement 10. AA increases open probability of WT but not K535 mutant TRPV4 channels. (a) Representative 15 s recordings obtained from cell-attached patches clamped at +80mV in HEK293 cells expressing WT, K535A or K535Q TRPV4 mutant channels in the presence of extracellular vehicle (DMSO 1:500) or Arachidonic Acid (AA 20 μM), as stated. Arrows indicates the closed state level. **(b)** Average open probability (NP_O) (expressed as the mean ± SEM) of WT and K535 mutant TRPV4 channels at the experimental conditions indicated above (vehicle, n=6; WT, n=11; K535A, n=7; K535Q, n=6). **P<0.01, *P<0.05 (Kruskal-Wallis followed by Dunn *post hoc* test).

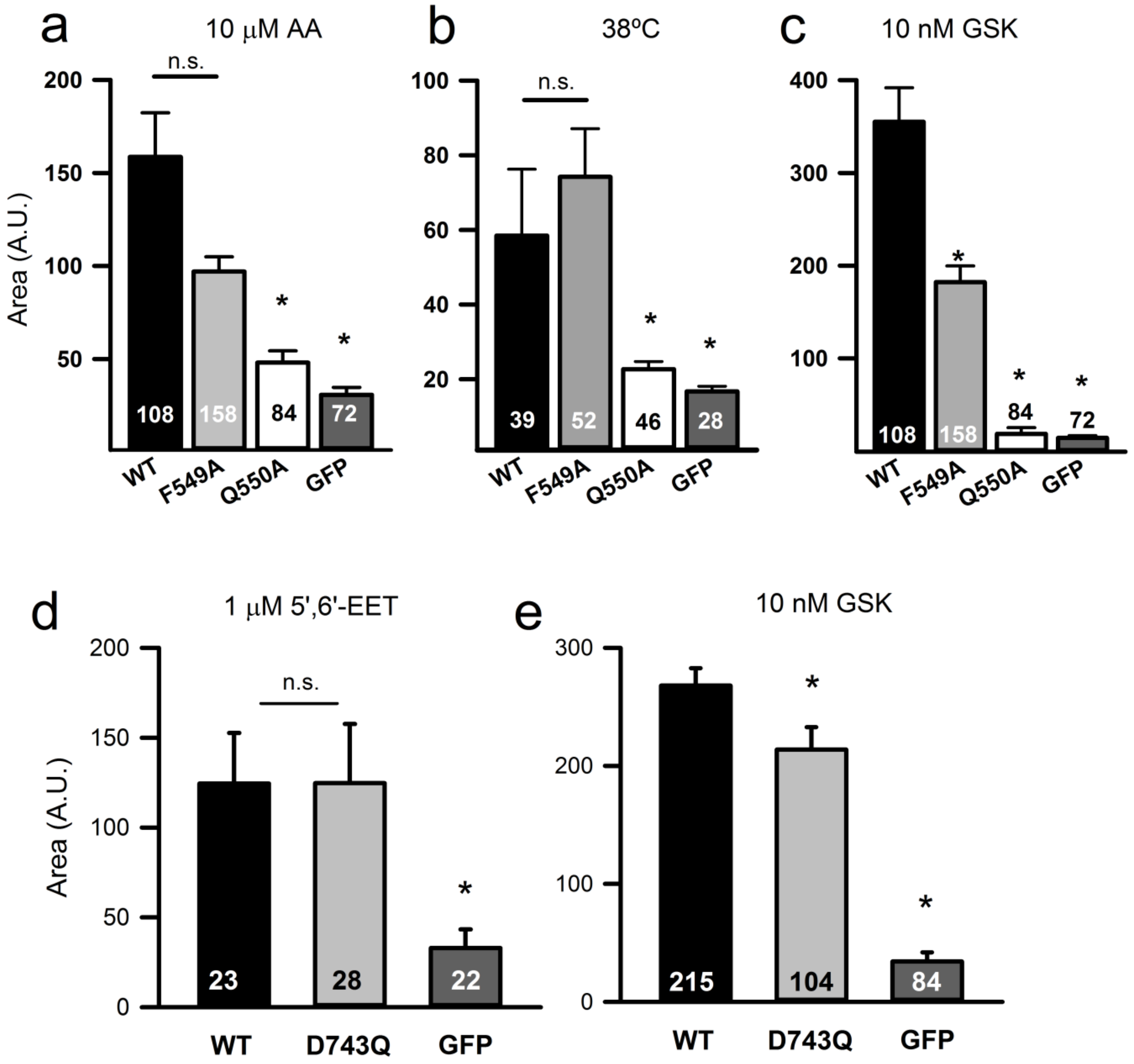

Figure supplement 11. Average $[Ca^{2+}]_i$ increases (area under the curve) obtained from cells expressing TRPV4-WT, TRPV4-F549A, TRPV4-Q550A, TRPV4-D743Q or GFP and exposed to 10 µM AA (**a**), 38ºC (**b**), 10 nM GSK1016790A (**c,e**) and 1 µM 5’,6’-EET (**d**). Numbers inside the bars indicate the number of cells analyzed. *P < 0.05 or not significant (n.s.) when compared with cells expressing TRPV4 WT channels (one way ANOVA followed by Bonferroni *post hoc* test).

S2-S3 linker

```
hTRPV4 (Q9HBA0) 530 KDLFMKKCPGVNSLFIDGSFQ 550
hTRPV1 (Q8NER1) 498      QRRPSMKTLFVDS    510
hTRPV2 (Q9Y5S1) 456      RRHVFIWISFIDSYFE 471
                       * ::    :    *:*.   :
```

S3

```
hTRPV4 (Q9HBA0) 551    LLYFIYSVLVIVSAALYLAGI 571
hTRPV1 (Q8NER1) 511 YSEMLFFLQSLFMLATVVLYF    531
hTRPV2 (Q9Y5S1) 472    ILFLFQALLTVVSQVLCFLAI 492
                      ::*:::  :::  :.:  .*  :   :
```

S4

```
hTRPV4 (Q9HBA0) 573 AYLAVMVFALVLGWMNALYFT 593
hTRPV1 (Q8NER1) 536 EYVASMVFSLALGWTNMLYYT 556
hTRPV2 (Q9Y5S1) 494 WYLPLLVSALVLGWLNLLYYT 514
                     *:   :*  :*.***  *  **:*
```

S4-S5 linker

```
hTRPV4 (Q9HBA0) 594 RGLKLTGTYSIMIQKILFKDLFR 616
hTRPV1 (Q8NER1) 557 RGFQQMGIYAVMIEK         571
hTRPV2 (Q9Y5S1) 515 RGFQHTGIYSVMIQKVILRDLLR 537
                    **::    *  *::**:*::::**  *
```

S5

```
hTRPV4 (Q9HBA0) 617         FLLVYLLFMIGYASALVSLLN 637
hTRPV1 (Q8NER1) 572 MILRDLCRFMFVYIVFLFGFSTAVVTLI  599
hTRPV2 (Q9Y5S1) 538         FLLIYLVFLFGFAVALVSLSQ 558
                    ::::**  **:::*::*::*::  *:*:*  :
```

S6

```
hTRPV4 (Q9HBA0) 691 YPVVFIILLVTYIILTFVLLL            711
hTRPV1 (Q8NER1) 659    VFIILLLAYVILTYILLLNMLIALMGETV 687
hTRPV2 (Q9Y5S1) 622     VLLLLLAYVLLTYILLLNMLI        642
                    :  :.::**::*::**::**********.***
```

Figure supplement 12. Comparison of human TRPV4, TRPV1 and TRPV2 sequences involved in binding of EET, capsaicin and lipids, respectively. Sequence alignment of S2-S3 linker, S3, S4, S4-S5 linker and S6 of human TRPV4, TRPV1 and TRPV2 channels. TRPV4 residues predicted to be within a distance of 3.5 Å of EET are shown in purple. TRPV1 residues involved in the binding of capsaicin are displayed in orange [59]. TRPV2

amino acids around the C-terminal region of S4 involved in lipid binding [58] (with some overlap with the binding-pocket for vanilloids at TRPV1) are depicted in blue. Closed arrows indicating those amino acids that are identical and open arrows pointing the conserved or semiconserved residues. “*” identical residues; “:” conserved substitutions (same amino acid group); “.” semi-conserved substitution (similar shapes).
